# Personality and Hypothalamic–Pituitary–Adrenal Axis in Older Men and Women

**DOI:** 10.3389/fpsyg.2020.00983

**Published:** 2020-06-05

**Authors:** Teresa Montoliu, Vanesa Hidalgo, Alicia Salvador

**Affiliations:** ^1^Laboratory of Social Cognitive Neuroscience, IDOCAL-Department of Psychobiology, Faculty of Psychology, University of Valencia, Valencia, Spain; ^2^IIS Aragón, Department of Psychology and Sociology, Area of Psychobiology, University of Zaragoza, Teruel, Spain

**Keywords:** neuroticism, conscientiousness, extraversion, hypothalamic–pituitary–adrenal axis, older adults

## Abstract

Personality has been related to health and mortality risk, which has created interest in the biological pathways that could explain this relationship. Although a dysregulation of the hypothalamic–pituitary–adrenal (HPA) axis has been associated with health outcomes and aging, few studies have explored the association between personality and HPA axis functioning in older adults. In addition, it has been suggested that sex could moderate the relationship between personality and HPA axis functioning. Thus, our aim was to analyze the relationship between the big five personality traits and the diurnal cortisol pattern in older adults, as well as sex differences in this relationship. To do so, 79 older people (40 men and 39 women) from 59 to 81 years old (*M* = 69.19, SD = 4.60) completed the NEO-Five-Factor Inventory (FFI) to measure neuroticism, conscientiousness, extraversion, openness, and agreeableness. Saliva samples were provided on three consecutive days (awakening; 15, 30, and 45 min post-awakening; and bedtime) in order to analyze the diurnal cortisol pattern and, specifically, two cortisol indexes: the cortisol awakening response (CAR) and the diurnal cortisol slope (DCS). Results showed that neuroticism and conscientiousness moderated the diurnal cortisol pattern. Thus, individuals with higher neuroticism and lower conscientiousness scores showed higher bedtime cortisol levels, suggesting a less healthy diurnal cortisol pattern. Regarding the cortisol indexes, higher neuroticism and lower conscientiousness were related to greater CAR and DCS. Sex moderated the association between extraversion and the DCS. Specifically, higher extraversion was related to a lower DCS only in women. Openness and agreeableness were not related to the diurnal cortisol pattern. In conclusion, our results show that in older adults, neuroticism is a vulnerability factor for HPA axis dysregulation, with possible adverse effects on health. By contrast, conscientiousness, and extraversion only in women, appear to be protective factors of HPA axis functioning, with potential beneficial effects on health.

## Introduction

The pace of the population’s aging around the world has increased dramatically in the past few decades. Although as people age, they are more likely to experience various health conditions, there is great heterogeneity in this process. Whereas some older adults maintain good physical and mental capacities, others experience a significant decline (World Health Organization [WHO], 2018). Therefore, it is important to identify vulnerability and protective factors in order to understand ways to improve health and longevity.

Personality traits have been associated with health, subjective well-being, and mortality risk during aging (Jerram and Coleman, 1999; Friedman et al., 2010; Weston et al., 2015). In addition, it has been suggested that the link between personality traits and health may be cumulative over time, and, therefore, personality could have a greater influence on disease in old age (Weston et al., 2015). Personality traits have been associated with aging-related diseases such as depression (Koorevaar et al., 2017), obesity (Sutin and Terracciano, 2016), heart disease, diabetes, and metabolic syndrome (Sutin et al., 2010; Mommersteeg and Pouwer, 2012), and cognitive impairment and dementia (Chapman et al., 2012; Low et al., 2013; Terracciano et al., 2014; Luchetti et al., 2016), among others. Specifically, in a longitudinal study carried out in a large sample of older adults, Weston et al. (2015) reported that greater neuroticism was a risk factor for disease onset, whereas conscientiousness and openness, and to a lesser degree, extraversion and agreeableness, were protective factors.

This evidence has created interest in studying the biological pathways that could explain the relationship between personality and health. Personality has been associated with hypothalamic–pituitary–adrenal axis (HPA axis) functioning (see review: Soliemanifar et al., 2018). The HPA axis is a neuroendocrine system that plays a key role in the stress response, whose end product in humans is glucocorticoid cortisol. The prefrontal cortex, hippocampus, and amygdala, with a high density of glucocorticoid receptors, are crucial in the regulation of this system (Fries et al., 2009), and it has been suggested that personality moderates an age-related decline in the volume of these brain regions (Jackson et al., 2011). In healthy humans, cortisol follows a diurnal rhythm, with a rapid increase upon awakening, peaking between 30 and 45 min post-awakening [conceptualized as the cortisol awakening response (CAR)] (Clow et al., 2004; Fries et al., 2009), and followed by a steady decrease throughout the day, reaching the lowest levels in the evening [the difference between awakening and evening cortisol is conceptualized as the diurnal cortisol slope (DCS)] (Adam et al., 2017). A dysregulation of the diurnal cortisol pattern, specifically a flattened DCS, has been associated with poorer mental and physical health (Adam et al., 2017) and chronic stress (Miller et al., 2007). However, both a larger and smaller CAR have been associated with poorer mental and physical health, although a larger CAR has mainly been related to life stress (Clow et al., 2004; Chida and Steptoe, 2009; Fries et al., 2009).

A dysregulation of HPA axis functioning has also been reported in aging, as a reduced CAR and flatter diurnal rhythm have been observed in older adults compared to young adults (Heaney et al., 2010). Moreover, although personality traits measure individual differences in relatively enduring patterns of thoughts, feelings, and behaviors, increased age is associated with changes in these traits. Specifically, conscientiousness and agreeableness tend to increase in older ages, whereas neuroticism and openness tend to decrease, and some dimensions of extraversion tend to increase, whereas others decrease (Roberts et al., 2006). Despite this, to our knowledge, only three studies have analyzed the association between the diurnal cortisol pattern and personality in older people (Gerritsen et al., 2009; Puig-Perez et al., 2016; Ouanes et al., 2017). In a previous study, we analyzed the association between neuroticism and extraversion, measured with the Eysenck Personality Questionnaire-Revised short form (EPQ-RS), and morning cortisol levels (Puig-Perez et al., 2016). We observed that higher neuroticism was related to lower overall morning cortisol concentrations [i.e., area under the curve with respect to the ground (AUCg)] and increased CAR only in women; however, extraversion was not associated with the AUCg or with the CAR (Puig-Perez et al., 2016). Gerritsen et al. (2009) also assessed neuroticism with the abbreviated subscale of the Dutch Personality Questionnaire, and they observed that higher neuroticism was related to higher evening cortisol levels, but not to post-awakening cortisol or diurnal cortisol variability. Of these three studies, only Ouanes et al. (2017) administered the NEO Five-Factor Inventory (NEO-FFI) to measure the big five personality traits (i.e., neuroticism, conscientiousness, extraversion, openness, and agreeableness), and they reported that higher neuroticism was related to a lower CAR, whereas lower extraversion and higher openness were associated with increased diurnal mean cortisol levels (AUCg). Moreover, although it has been reported that sex is an important moderator in the relationship between personality and HPA axis functioning (DeSoto and Salinas, 2015), only Puig-Perez et al. (2016) analyzed sex differences in the association between neuroticism and extraversion and morning cortisol levels, and they reported that higher neuroticism was related to a greater CAR only in women. Therefore, only a few studies have been carried out, but with methodological differences such as the use of different questionnaires to assess personality traits and different cortisol indexes used to measure HPA axis functioning, and sex differences in these associations have hardly been analyzed.

The aim of this study was to analyze the relationships between the big five personality traits (assessed with the NEO-FFI) and the diurnal cortisol pattern, considering the CAR and DCS indexes, in older people, as well as sex differences in these associations. Based on previous findings, we hypothesized that neuroticism and conscientiousness would be the personality traits most related to the diurnal cortisol pattern. Specifically, we expected to observe an association between a flatter DCS (Adam et al., 2017) and, mainly, higher scores on neuroticism, a personality trait considered to be a health risk factor, and lower scores on conscientiousness, which is considered a protective health factor (Lahey, 2009; Friedman and Kern, 2014). To a lesser degree, we also expected to observe an association between a flatter DCS and lower scores on other personality traits that are considered protective health factors (i.e., extraversion, openness, and agreeableness) (Weston et al., 2015). Because both larger and smaller CAR have been related to worse health (Clow et al., 2004; Chida and Steptoe, 2009; Fries et al., 2009), we were not able to predict the direction of the association between personality traits and the CAR. However, as a larger CAR has been associated with life stress and worrying (Chida and Steptoe, 2009; Fries et al., 2009), we hypothesized that greater neuroticism (characterized by emotional instability and distress proneness) would be related to a larger CAR. In addition, we expected to observe an association between higher neuroticism and a dysregulation of the HPA axis (greater CAR) mainly in women, as reported in Puig-Perez et al. (2016).

## Materials and Methods

### Participants

A final sample of 79 people (40 men and 39 women) ranging in age from 59 to 81 years (*M* = 69.19, SD = 4.60) participated in the research. Participants belonged to a 4-year follow-up study and were initially recruited from a study program at the University of Valencia (Spain) for people over 55 years of age. Exclusion criteria were: smoking more than 10 cigarettes a day, abuse of alcohol (no more than 20 g/day for women and 30 g/day for men) or other drugs, having been under general anesthesia and/or the presence of a stressful life event in the past year (such as the death of a loved one, an accident, an important change in their habits, such as retirement, or any other event that they subjectively felt had affected them in a significant way), and illness (severe psychiatric or endocrine disorders) or medication (such as glucocorticoids, anticonvulsants, and opioids) that could influence hormonal levels, as reported in Nicolson (2008). Of the 79 participants, 15 (19%) took anxiety or sleep medication. As Nicolson (2008) suggested, we made sure that this medication did not influence cortisol levels. Therefore, we compared the CAR and the DCS indexes in participants who took this medication and those who did not, and no significant differences were found (all *p* ≥ 0.377). In addition, anxiety/sleep medication intake was included as a covariate in the regression models. All the women were postmenopausal and had their last menstrual period more than 3 years before the testing time, and none of the participants scored below 27 on the MEC (Spanish version of the Mini-Mental Status Examination; Lobo et al., 1999), indicating the absence of cognitive impairment.

### Procedure

Participants who agreed to participate were asked to attend one session that took place in the Laboratory of Social Cognitive Neuroscience at the University of Valencia. They were asked to fill out the Spanish version (Costa and McCrae, 1999) of the NEO-FFI (Costa and McCrae, 1992). In addition, participants reported their subjective socioeconomic status (SES). For this purpose, they were given a drawing of a ladder with 10 rungs and asked to place an X on the rung that best represented where they thought they stood in society. The top of the ladder represented those with more money, more education, and better jobs, whereas the bottom represented those with less money, less education, and worse jobs (Adler et al., 2000). In addition, body mass index (BMI) was measured, and participants provided a total of 15 saliva samples using salivettes (Sarstedt, Nümbrecht, Germany). They were instructed to keep the cotton swab in their mouths for exactly 2 min, not chew the cotton, and move the swab around in a circular pattern to collect saliva from all the salivary glands. These saliva samples were collected at home immediately after awakening; 15, 30, and 45 min post-awakening; and immediately before bedtime on three consecutive days. Participants stored the saliva samples in the refrigerator until they were delivered to the laboratory.

All the participants provided written informed consent to participate in the study, which was conducted in accordance with the 64th WMA Declaration of Helsinki (October 2013) and approved by the Research Ethics Committee of the University of Valencia.

#### NEO-Five Factor Inventory

The Spanish version (Costa and McCrae, 1999) of the NEO-FFI (Costa and McCrae, 1992) was used to measure the Big Five personality traits. The NEO-FFI consists of 60 items that measure neuroticism, conscientiousness, extraversion, openness, and agreeableness, with 12 items for each trait. The items are answered on 5-point scales (ranging from 0 to 4), and higher scores indicate a higher degree of the trait. The internal reliabilities for the subscales in the present study were good, with the following Cronbach’s alphas: 0.84 (neuroticism), 0.79 (conscientiousness), 0.82 (extraversion), 0.70 (openness), and 0.79 (agreeableness).

#### Salivary Cortisol Determination

After participants returned the saliva samples to the laboratory, the samples were kept in the refrigerator until they were centrifuged at 3,000 rpm for 5 min, resulting in a clear supernatant of low viscosity that was stored at −80°C until the analyses of the salivary cortisol levels. HPA axis activity was measured by analyzing the salivary cortisol levels. Salivary cortisol concentrations were determined in duplicate with the salivary cortisol enzyme immunoassay kit from Salimetrics (Newmarket, United Kingdom). Assay sensitivity was <0.007 μg/dl. For each subject, all the samples were analyzed in the same trial. The inter- and intra- assay variation coefficients were all below 10%. Cortisol levels were expressed in no/L.

### Statistical Analyses

Participants’ characteristics were described using means (*M*) [standard deviation (SD)] for the total sample and for men and women separately. To investigate sex differences in age, subjective SES, BMI, and personality traits, independent sample Student *t* tests were performed. Cohen’s *d* was calculated to obtain the effect size.

Before the statistical analyses were performed, cortisol data were checked for normal distribution and homogeneity of variance using Kolmogorov–Smirnov and Levene’s test. These analyses revealed significant deviations in cortisol values; therefore, cortisol data were logarithm 10 (Log10) transformed. For each of the 3 days, we obtained two cortisol indexes: (i) the CAR, measured as the area under the curve with respect to the increase (AUCi) (Pruessner et al., 2003), and (ii) the DCS, calculated as bedtime cortisol minus awakening cortisol (Adam et al., 2017). The CAR and DCS indexes for the 3 days were averaged (correlation analyses showed all *p* ≤ 0.001, and *p* ≤ 0.002, respectively). One woman scored + 3 SD from the mean on the CAR, and she was excluded from the analyses.

First, Pearson’s correlations were performed to analyze the association between the sociodemographic variables (age, sex, SES, BMI), awakening hour, anxiety or sleep medication, personality traits (neuroticism, conscientiousness, extraversion, openness, or agreeableness), and cortisol indexes (CAR and DCS).

In order to explore the diurnal cortisol pattern, an ANOVA for repeated measures was performed, with Day (1, 2, and 3) and Time (awakening; 15, 30, and 45 min post-awakening; and bedtime cortisol) as within-subject factors. After that, for each personality trait (neuroticism, conscientiousness, extraversion, openness, or agreeableness), participants were categorized according to whether they obtained high (above the median) or low (below the median) scores. Different ANOVAs for repeated measures were performed, with Day and Time as within-subject factors and one personality trait as a between-subject factor. Greenhouse–Geisser was used when the requirement of sphericity was violated. *Post hoc* planned comparisons were performed using Bonferroni adjustments for the *p* values.

Then, to investigate whether there was an association between personality traits (as continuous variables) and the CAR and DCS indexes, separate linear regression analyses were performed with each cortisol index as a dependent variable. We conducted hierarchical analyses, including the covariates (age, sex, SES, BMI, awakening time, and anxiety or sleep medication) in step 1 following stepwise analysis, and one personality trait in step 2. Finally, in order to analyze whether there were sex differences in the association between personality traits and the CAR and DCS indexes, moderated regression analyses were computed. To do so, the PROCESS macro in SPSS (Model 1) was used with 5,000 bootstrapped samples, including one personality trait as the independent variable, one cortisol index as the dependent variable, sex as moderator, and the covariates.

To perform these statistical analyses, version 25.0 of SPSS was used. All *p* values were two-tailed, and the level of significance was taken as *p* ≤ 0.05.

## Results

### Participants’ Characteristics and Descriptives

There were no significant differences between men and women in age or BMI (all *p* ≥ 0.581), but men showed a significantly higher SES than women [*t* (76) = −3.149, *p* = 0.002, *d* = −0.675]. Regarding the personality traits, women scored significantly higher on neuroticism [*t* (77) = 2.704, *p* = 0.008, *d* = 0.585] and agreeableness [*t* (77) = 2.185, *p* = 0.032, *d* = 0.480] than men. There were no sex differences in conscientiousness, extraversion, or openness scores (all *p* ≥ 0.316) (Table 1).

**TABLE 1:** Characteristics of the study population for the total sample and for men and women separately.

	Total (*N* = 79)	Men (*N* = 40)	Women (*N* = 39)	*t*	*gl*	*p*	95% CI	*d*
	*M* (SD)	*M* (SD)	*M* (SD)					
Age	69.19 (4.60)	69.48 (4.84)	68.90 (4.40)	−0.555	77	0.581	−2.652, 1.496	−0.125
SES	5.74 (1.31)	6.18 (1.28)	5.29 (1.19)	−3.149	76	0.002	−1.446, −0.326	−0.675
BMI	27.48 (3.65)	27.53 (2.58)	27.43 (4.53)	−0.112	59.997	0.904	−1.769, 1.566	−0.027
Neuroticism	16.20 (7.01)	14.17 (6.44)	18.28 (7.05)	2.704	77	0.008	1.082, 7.131	0.585
Conscientiousness	33.54 (5.62)	34.17 (5.83)	32.89 (5.41)	−1.009	77	0.316	−3.799, 1.244	−0.226
Extraversion	28.84 (6.75)	28.20 (7.32)	29.51 (6.13)	0.863	77	0.391	−1.717, 4.343	0.194
Openness	29.73 (5.60)	29.12 (6.06)	30.35 (5.09)	0.978	77	0.331	−1.278, 3.746	0.220
Agreeableness	32.03 (5.85)	30.65 (5.90)	33.46 (5.51)	2.185	77	0.032	0.249, 5.373	0.480

*SES, socioeconomic status; BMI, body mass index. Sex differences were analyzed with Student *t* tests, and effect sizes with Cohen’s *d*.*

In addition, Pearson’s correlation analyses showed that individuals with higher neuroticism (*r* = −0.24, *p* = 0.037) and conscientiousness (*r* = 0.25, *p* = 0.031) scores reported higher SES, whereas individuals with higher neuroticism scores showed higher BMI (*r* = 0.24, *p* = 0.036). Moreover, awakening hour was negatively related to age (*r* = −0.26, *p* = 0.025), agreeableness (*r* = −0.25, *p* = 0.030), and the DCS (*r* = −0.28, *p* = 0.016). Furthermore, higher neuroticism scores were associated with lower conscientiousness (*r* = −0.46, *p* ≤ 0.001) and extraversion (*r* = −0.42, *p* ≤ 0.001) scores. In turn, higher conscientiousness was related to higher extraversion (*r* = 0.28, *p* = 0.014) scores. Moreover, higher neuroticism scores were related to higher CAR (*r* = 0.28, *p* = 0.014) and DCS (*r* = 0.37, *p* = 0.001), whereas lower conscientiousness scores were related to higher DCS (*r* = −0.35, *p* = 0.002). Finally, the CAR was positively related to the DCS (*r* = 0.28, *p* = 0.015) (Table 2).

**TABLE 2:** Pearson’s correlations between sociodemographic variables, personality traits, and cortisol indexes.

	1	2	3	4	5	6	7	8	9	10	11	12
Age (1)	–	–	–	–	–	–	–	–	–	–	–	–
Sex (2)	0.06	–	–	–	–	–	–	–	–	–	–	–
SES (3)	0.01	0.34**	–	–	–	–	–	–	–	–	–	–
IMC (4)	0.02	−0.00	−0.22^#^	–	–	–	–	–	–	–	–	–
Awakening hour (5)	−0.26*	0.01	−0.00	−0.00	–	–	–	–	–	–	–	–
Medication (6)	−0.05	−0.11	−0.03	0.03	0.22^#^	–	–	–	–	–	–	–
Neuroticism (7)	−0.03	−0.29**	−0.24*	0.24*	−0.20^#^	−0.14	–	–	–	–	–	–
Conscientiousness (8)	0.06	0.13	0.25*	−0.22^#^	0.04	0.22^#^	−0.46**	–	–	–	–	–
Extraversion (9)	−0.11	−0.10	0.12	−0.01	0.08	0.16	−0.42**	0.28*	–	–	–	–
Openness (10)	−0.05	−0.10	−0.05	−0.03	−0.12	0.11	0.09	0.03	0.17	–	–	–
Agreeableness (11)	0.05	−0.24*	−0.21^#^	−0.07	−0.25*	0.06	−0.14	0.15	0.13	0.19^#^	–	–
CAR (12)	0.01	−0.17	−0.07	0.00	−0.17	−0.10	0.28*	−0.19^#^	−0.16	−0.12	0.05	–
DCS (13)	0.11	−0.09	−0.03	0.17	−0.28*	0.02	0.37**	−0.35**	−0.11	−0.12	−0.10	0.28*

SES, socioeconomic status; BMI, body mass index; CAR, cortisol awakening response; DCS, diurnal cortisol slope. ^#^*p* < 0.10, *p < 0.05, **p < 0.01.

### Personality Differences in the Diurnal Cortisol Pattern

**FIGURE 1:**
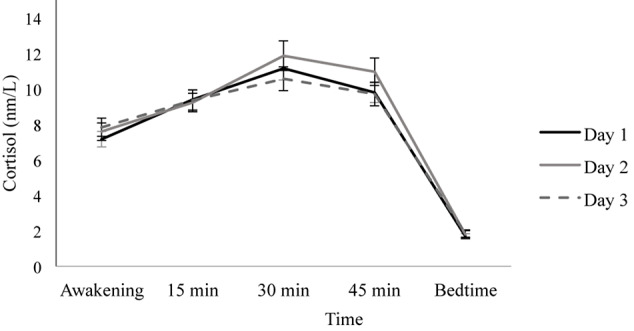
Diurnal cortisol pattern on three consecutive days. Depicted values are means, and error bars represent SEM.

Overall, the repeated-measures ANOVA did not show a main effect of Day [*F*(1.797, 122.216) = 0.277, *p* = 0.735, ηp^2^ = 0.004], but a significant main effect of Time was found [*F*(2.007, 136.491) = 358.270, *p* ≤ 0.001, ηp^2^ = 0.840]. As Figure 1 shows, participants presented the CAR because their cortisol levels increased from awakening to 15 and 30 min post-awakening, and then they decreased slightly at 45 min post-awakening, reaching the lowest levels before bedtime (all *p* < 0.001).

**FIGURE 2:**
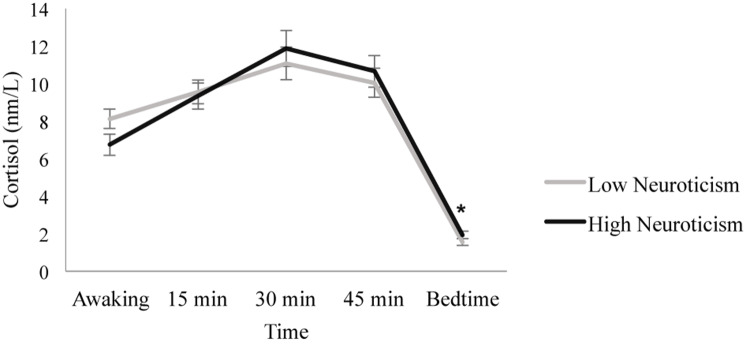
Diurnal cortisol pattern in individuals with high and low neuroticism scores. Individuals with low neuroticism scores showed lower bedtime cortisol levels than individuals with high neuroticism scores (*p = 0.044). Depicted values are means, and error bars represent SEM.

When introducing neuroticism as a between-subject factor, the Day factor [*F*(1.797, 120.405) = 0.296, *p* = 0.721, ηp^2^ = 0.004] and the Day × Neuroticism interaction [*F*(1.797, 120.405) = 0.969, *p* = 0.375, ηp^2^ = 0.014] were not significant. However, significant main effects of Time [*F*(1.938, 129.849) = 368.415, *p* ≤ 0.001, ηp^2^ = 0.846] and the Time × Neuroticism interaction [*F*(1.938, 129.849) = 4.572, *p* = 0.013, ηp^2^ = 0.064] were found. *Post hoc* analyses revealed no significant differences between the individuals with high and low neuroticism scores on awakening, 15, 30, or 45 min post-awakening cortisol (all *p* ≥ 0.108), but individuals with low neuroticism scores showed lower bedtime cortisol levels than individuals with high neuroticism scores [*F*(1, 67) = 4.213, *p* = 0.044, ηp^2^ = 0.059]. In addition, in individuals with low neuroticism scores, cortisol levels increased from awakening to 15 min (*p* = 0.009), but there were no significant differences between the 15 and 30 min cortisol levels (*p* = 0.192). Then, cortisol levels decreased from 30 to 45 min post-awakening (*p* = 0.015) and from 45 min post-awakening to bedtime (*p* ≤ 0.001). Similarly, in individuals with high neuroticism scores, cortisol levels increased from awakening to 15 min (*p* ≤ 0.001) and from 15 to 30 min post-awakening (*p* ≤ 0.001), and they decreased from 30 to 45 min post-awakening (*p* = 0.012) and from 45 min post-awakening to bedtime (*p* ≤ 0.001) (Figure 2).

**FIGURE 3:**
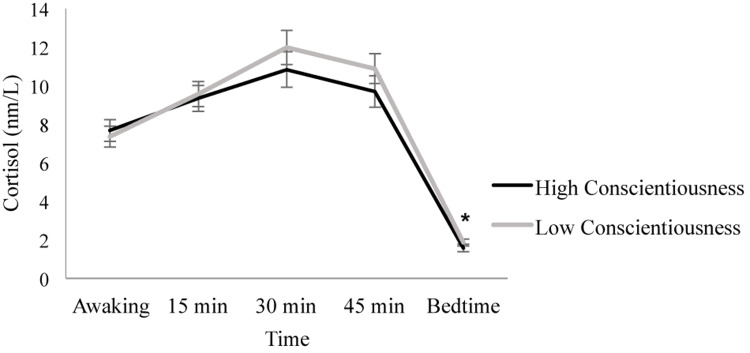
Diurnal cortisol pattern in individuals with high and low conscientiousness scores. Individuals with low conscientiousness scores showed higher bedtime cortisol levels than individuals with high conscientiousness scores (*p = 0.037). Depicted values are means, and error bars represent SEM.

When conscientiousness was introduced as a between-subject factor, results showed that Day [*F*(1.780, 119.264) = 0.232, *p* = 0.767, ηp^2^ = 0.003] and the Day × Conscientiousness interaction [*F*(1.780, 119.264) = 1.032, *p* = 0.352, ηp^2^ = 0.015] were not significant. In contrast, the Time factor [*F*(1.990, 133.330) = 371.202, *p* ≤ 0.001, ηp^2^ = 0.847] and the Time × Conscientiousness interaction [*F*(1.990, 133.330) = 3.172, *p* = 0.045, ηp^2^ = 0.045] were significant. *Post hoc* analyses showed no significant differences between the individuals with high and low conscientiousness scores in all the cortisol samples (all *p* ≥ 0.344), except the bedtime sample, where individuals with low conscientiousness scores showed higher cortisol levels than individuals with high conscientiousness scores [*F*(1, 67) = 4.553, *p* = 0.037, ηp^2^ = 0.064]. Furthermore, the individuals with low conscientiousness scores increased their cortisol levels from awakening to 15 min (*p* ≤ 0.001) and from 15 to 30 min after awakening (*p* ≤ 0.001). Then, cortisol levels decreased from 30 to 45 min post-awakening (*p* = 0.005) and from 45 min post-awakening to bedtime (*p* ≤ 0.001). Similarly, in individuals with high conscientiousness scores, cortisol levels increased from awakening to 15 min (*p* = 0.002), but they were similar 15 and 30 min after awakening (*p* = 0.467), and they decreased from 30 to 45 min post-awakening (*p* = 0.038) and from 45 min post-awakening to bedtime (*p* ≤ 0.001) (Figure 3).

However, when extraversion, openness, and agreeableness were introduced as between-subject factors, no main effect of Day (all *p* ≥ 0.451) or the Day × Extraversion, Day × Openness, or Day × Agreeableness interaction (all *p* ≥ 0.076) was observed. Although the Time factor was significant (all *p* ≤ 0.001), its interaction with each personality trait was not significant (all *p* ≥ 0.168).

### Relationship Between Personality and Cortisol Indexes

Linear regression analyses showed that the CAR was positively related to neuroticism (*B* = 0.308, *p* = 0.008) and negatively related to conscientiousness (*B* = −0.254, *p* = 0.029), but not to the rest of the personality traits (all *p* ≥ 0.098), and all the covariates were excluded from the model. Moreover, the DCS was positively related to neuroticism (*B* = 0.312, *p* = 0.006) and negatively related to conscientiousness (*B* = −0.338, *p* = 0.002), but not to the rest of the personality traits (all *p* ≥ 0.164). Only the awakening hour contributed to the association between the personality traits and the DCS (*ΔR^2^* = 0.083, *B* = −0.338, *p* = 0.002), whereas the rest of the covariates were excluded from the model (Table 3).

**TABLE 3:** Regression analyses with personality traits as predictors and the cortisol indexes as dependent variables, adjusted for age, sex, SES, BMI, awakening hour, and sleep/anxiety medication.

	CAR			DCS		
	*ΔR*^2^	Beta	*p*	*ΔR*^2^	Beta	*p*
Awakening hour	–	–	–	–	−0.225*	0.046
Neuroticism	0.095	0.308**	0.008	0.094	0.312**	0.006
Awakening hour	–	–	–	–	−0.272*	0.013
Conscientiousness	0.064	−0.254*	0.029	0.114	−0.338**	0.002
Awakening hour	–	–	–	–	−0.280*	0.017
Extraversion	0.038	−0.194	0.098	0.007	−0.081	0.480
Awakening hour	–	–	–	–	−0.302*	0.010
Openness	0.012	−0.111	0.348	0.023	−0.158	0.167
Awakening hour	–	–	–	–	−0.326**	0.006
Agreeableness	0.000	0.019	0.871	0.024	−0.163	0.164

CAR, cortisol awakening response; DCS, diurnal cortisol slope. *ΔR*: change *R*^2^. All covariates were excluded from the model, except awakening hour, which contributed to the relationship between personality traits and the DCS. *p < 0.05, **p < 0.01.

Finally, sex only significantly moderated the association between extraversion and the DCS (Δ*R*^2^ = 0.068, *p* = 0.023, CI 95% 0.076, 1.003). Specifically, results showed a significant negative relationship between extraversion and the DCS in women (*B* = −0.439, SE = 0.179, *p* = 0.016, CI 95% −0.797, −0.082) but not in men (*B* = 0.100, SE = 0.148, *p* = 0.502, CI 95% −0.196, 0.396).

## Discussion

Our results showed that neuroticism and conscientiousness moderated the diurnal cortisol pattern. Specifically, individuals with higher neuroticism and lower conscientiousness scores showed higher bedtime cortisol levels, indicating a less healthy profile. Similarly, higher neuroticism and lower conscientiousness were associated with a greater CAR and a flattened DCS, which also shows a less healthy diurnal cortisol pattern. Sex only moderated the association between extraversion and the DCS. Specifically, higher extraversion was related to a steeper DCS only in women, indicating a healthier profile. Openness and agreeableness were not related to the diurnal cortisol pattern.

### Neuroticism and Conscientiousness, and Hypothalamic–Pituitary–Adrenal Axis

Individuals who score higher on neuroticism tend to perceive more stressors and respond with intense emotional reactions, and so this personality trait correlates highly with chronic stress, negative feelings, and anxiety (Lahey, 2009). In addition, stress and worrying have been related to an increased CAR (Chida and Steptoe, 2009; Fries et al., 2009), which would support our finding that greater neuroticism was related to a larger CAR. We previously observed that higher neuroticism was related to a larger CAR in women in the same sample of older adults, but assessed 4 years earlier and with a different personality questionnaire (EPQ-R) (Puig-Perez et al., 2016). Therefore, our results replicated these previous findings, confirming an association between neuroticism and a larger CAR and, thus, less healthy HPA axis functioning in older adults. By contrast, Ouanes et al. (2017) explored the association between the big five personality traits (assessed with the NEO-FFI-R) and the CAR and diurnal mean cortisol (AUCg) in older people, and they reported a negative association between neuroticism and the CAR. However, although Ouanes et al.’s (2017) study was carried out in a larger sample, saliva samples were collected on a single day, and the CAR was calculated with saliva samples measured at two time points (awakening and 30 min after wakening), whereas in our study, the CAR was calculated with saliva samples at four time points (awakening and 15, 30, and 45 min post-awakening) on three consecutive days.

Neuroticism has been considered an important predictor not only of mental health problems, such as anxiety and depression, but also of physical health and mortality risk (see reviews: Lahey, 2009; Friedman and Kern, 2014). Supporting this evidence, our results showed that individuals with greater neuroticism showed a greater DCS (i.e., a smaller decrease in cortisol levels throughout the day resulting in a flattened diurnal cortisol slope), which has been related to worse physical and mental health (Adam et al., 2017) and chronic stress (Miller et al., 2007). In addition, our results also showed that individuals with higher neuroticism scores showed higher bedtime cortisol levels, which would lead to a flattened DCS. In line with our results, Gerritsen et al. (2009), in a large sample of older people, reported that greater neuroticism was related to higher bedtime cortisol levels in participants below 75 years old. However, contrary to our results, Gerritsen et al. (2009) failed to observe an association between neuroticism and the DCS. Nevertheless, this discrepancy could be due to methodological differences. For example, in Gerritsen et al.’s (2009) study, neuroticism was assessed with a different questionnaire, and the DCS was measured as the difference between the 30-min peak and bedtime salivary cortisol measured on a single day.

The evidence that conscientiousness has been related to better coping and emotion regulation abilities (see review: Friedman and Kern, 2014), along with the fact that a greater CAR has been related to stress (Chida and Steptoe, 2009; Fries et al., 2009), would support the association between greater conscientiousness and a lower CAR observed in this study. In addition, there is growing evidence that conscientiousness is a strong predictor of health and longevity possibly because conscientious individuals tend to engage in healthier behaviors such as smoking less, moderate alcohol consumption, physical exercise, and eating healthier food. They also tend to have healthier friendships, more stable marriages, a better education, more meaningful careers, and higher incomes (see review: Friedman and Kern, 2014). This evidence would explain our results showing that individuals with greater conscientiousness had lower bedtime cortisol levels and a lower DCS (a greater decrease in cortisol levels throughout the day, resulting in a steeper DCS), which has been related to better physical and mental health (Adam et al., 2017). Supporting our results, in a recent study in a large sample of older adults, Steptoe et al. (2017) found that greater conscientiousness was related to low hair cortisol concentrations, which is an indicator of tonic cortisol output over several weeks, suggesting healthier HPA axis functioning. However, unlike in our study, in a larger sample, Ouanes et al. (2017) did not observe an association between conscientiousness and the diurnal cortisol pattern (CAR and AUCg), but they only considered two saliva samples on a single day.

Of the big five personality traits, neuroticism and conscientiousness have shown the strongest links with health (see reviews: Lahey, 2009; Friedman and Kern, 2014). In this line, our results showed that, in older people, lower neuroticism and greater conscientiousness were related to a healthier diurnal cortisol rhythm. Supporting our results, in a study carried out in middle-aged and older healthy adults, greater neuroticism and lower conscientiousness were related to a greater decline in prefrontal and medial temporal region volumes (Jackson et al., 2011), brain areas that contribute to HPA axis regulation functioning (Fries et al., 2009). Other studies also analyzed the association between neuroticism and conscientiousness and the diurnal cortisol pattern but in samples with a wide age range, including both young and older people. Similar to our findings, Bogg and Slatcher (2015), in a sample with a broad age range (25–75 years old), observed that higher conscientiousness was related to a steeper (i.e., healthier) DSC, but, unlike in our study, they failed to observe an association between neuroticism and the DCS. In addition, other studies failed to find an association between neuroticism and conscientiousness and the CAR (Van Santen et al., 2011; Hill et al., 2013; Bogg and Slatcher, 2015). However, it has been suggested that the influence of personality traits on health may accumulate over time, and, consequently, personality may have a greater influence on disease in old age (Weston et al., 2015). This could explain why the association between neuroticism and conscientiousness and a less healthy diurnal cortisol pattern (considering the CAR and DCS indexes) is observed in older adults, but not in samples that also include younger participants.

Finally, although we expected to find an association between higher neuroticism and a greater CAR in women (Puig-Perez et al., 2016), we failed to observe a moderating effect of sex on the association between neuroticism and conscientiousness and the CAR and DSC. Therefore, our results suggest that, in older people, higher neuroticism and lower conscientiousness are related to less healthy HPA axis functioning in both men and women.

### Extraversion and Hypothalamic–Pituitary–Adrenal Axis

We failed to observe an association between extraversion and the CAR, in line with previous studies in older people (Puig-Perez et al., 2016; Ouanes et al., 2017). However, in studies with samples that included young and older people, one study observed an association between greater extraversion and a lower CAR (Van Santen et al., 2011), whereas others did not (Munafò et al., 2006; Hill et al., 2013). Moreover, in older people, Ouanes et al. (2017) observed that higher extraversion was related to lower diurnal mean cortisol (AUCg), suggesting an association between extraversion and a healthier diurnal cortisol rhythm. Interestingly, although we did not observe an association between extraversion and the DCS, we observed a moderating effect of sex on this association. Specifically, we observed that higher extraversion was related to a lower DCS, indicating a healthier diurnal cortisol rhythm, in women, but not in men. Therefore, sex differences could explain mixed findings reported on the association between extraversion and HPA axis functioning. Moreover, although extraversion has been linked to positive affect, this personality trait has also been associated with both positive (diet and exercise) and negative (alcohol and smoking) health behaviors (Booth-Kewley and Vickers, 1994), which could also explain the inconsistent findings reported.

### Openness and Agreeableness, and Hypothalamic–Pituitary–Adrenal Axis

A previous study in a large sample of older adults failed to observe an association between agreeableness and the diurnal cortisol pattern (Ouanes et al., 2017). However, Ouanes et al. (2017) reported that greater openness was related to higher diurnal mean cortisol (AUCg) measured on a single day. By contrast, we failed to observe significant associations between openness and agreeableness and the diurnal cortisol pattern, coinciding with studies that included both young and older participants (Van Santen et al., 2011; Hill et al., 2013). Therefore, although openness and agreeableness have been considered protective health factors in older adults (Weston et al., 2015), our study suggests that these personality traits are not associated with HPA axis functioning.

### Practical Implications

Our results showed that, in older adults, personality traits are associated with HPA axis functioning, which in turn has been related to physical and mental health outcomes (Adam et al., 2017). Therefore, this evidence can help to deepen the knowledge and create more comprehensive models of the relationship between personality and health. Future research in this field could analyze the mediating role of the HPA axis in these relationships between personality traits and specific health diseases. Moreover, research on HPA axis functioning could consider personality traits as possible moderators/confounders to take into account. Ultimately, the goal of research on personality and health is to create effective interventions to promote health. Although personality is a relatively stable trait, some related cognitive–behavioral patterns can be modified, such as healthy habits (i.e., diet and physical exercise), stress and anxiety management, or the development of social networks. Therefore, one of the biggest challenges of health psychology is to understand and develop interventions at the individual and social levels to help individuals to adhere to healthy pathways in order to improve health and well-being (Friedman and Kern, 2014). A possible innovative and inexpensive approach would be to use personality scales in a large number of individuals as a screening tool for the early detection of higher risk individuals (i.e., higher neuroticism) in early stages when preventive interventions can be more helpful (Lahey, 2009). This could be especially relevant and useful for a vulnerable group such as older people.

### Limitations and Strengths

Some limitations should be considered. First, the correlational nature of the results means that we cannot claim causal relationships. Moreover, a larger sample size would allow us to increase the statistical power in detecting sex differences in the associations between personality traits and the cortisol indexes. However, this study also has some strengths, such as the measurement of the diurnal cortisol pattern on three consecutive days and the inclusion of the big five personality traits. Another strength of our study is the fact that we only included older people because results on the association between personality and HPA axis functioning at younger ages could not be generalized to this age group. However, it would be valuable for future research to compare this association in different age groups (young, middle-age, and older adults).

### Conclusion

In conclusion, our results show that, in older adults, neuroticism is a vulnerability factor for HPA axis dysregulation, with possible adverse effects on health. By contrast, conscientiousness is a protective factor of HPA axis functioning, with potential beneficial effects on health. Moreover, extraversion appears to be a protective factor of HPA axis functioning only in women. Finally, agreeableness and openness are not related to HPA axis functioning, at least with regard to the diurnal cortisol pattern and based on the cortisol indexes considered in the present study.

## Data Availability Statement

The datasets generated for this study are available on request to the corresponding author.

## Ethics Statement

The studies involving human participants were reviewed and approved by Research Ethics Committee of the University of Valencia. The patients/participants provided their written informed consent to participate in this study.

## Author Contributions

All authors made substantial contributions. TM conceived and designed the analysis, collected the data, contributed data or analysis tools, performed the analyses, and wrote the manuscript. VH conceived and designed the analysis, performed the analyses, and reviewed the manuscript. AS conceived and designed the analyses and reviewed the manuscript.

## Conflict of Interest

The authors declare that the research was conducted in the absence of any commercial or financial relationships that could be construed as a potential conflict of interest.
